# The Role of Amino Acids in the Formation of Aroma-Active Compounds during Shrimp Hot Air Drying by GC-MS and GC-IMS

**DOI:** 10.3390/foods11203264

**Published:** 2022-10-19

**Authors:** Zewei Zhang, Hongwu Ji, Di Zhang, Shucheng Liu, Xiaoshan Zheng

**Affiliations:** 1 Guangdong Provincial Key Laboratory of Aquatic Product Processing and Safety, College of Food Science and Technology, Guangdong Ocean University, Zhanjiang 524088, China; 2 Guangdong Provincial Engineering Technology Research Center of Seafood, College of Food Science and Technology, Guangdong Ocean University, Zhanjiang 524088, China; 3 Guangdong Province Engineering Laboratory for Marine Biological Products, College of Food Science and Technology, Guangdong Ocean University, Zhanjiang 524088, China; 4 Key Laboratory of Advanced Processing of Aquatic Product of Guangdong Higher Education Institution, College of Food Science and Technology, Guangdong Ocean University, Zhanjiang 524088, China; 5 Collaborative Innovation Center of Seafood Deep Processing, Dalian Polytechnic University, Dalian 116034, China

**Keywords:** shrimp, amino acids, aroma-active compounds, gas chromatography-mass spectrometry, gas chromatography-ion mobility spectrometry

## Abstract

In the present paper, the role of amino acids of *Penaeus vannamei* was investigated in the formation of volatile substances during drying. The variations in volatile substances among samples with different moisture contents (raw, 45%, 30%, 15%, and 5%) were obtained by gas chromatography-ion mobility spectrometry (GC-IMS) and gas chromatography-mass spectrometry (GC-MS). The amino acid contents of the above samples were measured by the amino acid automatic analyzer. Correlation between pyrazines and the various amino acid contents was analyzed by the Pearson correlation coefficient. Their correlation was verified by conducting addition assays. The types and contents of volatile components increased significantly in samples with moisture contents between 30% and 5%. The most obvious increases in the type, content and odor activity value of pyrazines were observed in this range. Basic amino acids (Arg, Lys, and His) had a strong correlation with the formation of pyrazines. Addition assays verified that the addition of Arg and Lys increased the content of pyrazines in shrimp after drying.

## 1. Introduction

The aroma of dried products plays an important role in their quality evaluation and acceptance by consumers [1]. Dried shrimp products are favored by consumers for their pleasant aroma, which results from the combined action of a large number of volatile components [2]. Volatile compounds have been identified in the Argentine red shrimp [3], spotted shrimp [4], white shrimp [5,6], and whiteleg shrimp [2] after thermal processing. Among these identified compounds, aroma-active compounds (AACs) that contribute significantly to the overall aroma of penaeid shrimp mainly include 3-ethyl-2,5-dimethylpyrazine, 2-ethyl-5-methylpyrazine, 2,5-dimethylpyrazine, 2-acetyl-1-pyrroline, 1-octen-3-ol, benzaldehyde, 3-methylbutyraldehyde, 2-methylbutyraldehyde, 3-(methylthio)propionaldehyde, and trimethylamine. The sources of AACs include the shrimp itself and the formation by reactions, such as the thermal degradation of lipids and the Maillard reaction, among others [7]. Moisture content and precursor types are two important factors affecting the formation of these compounds. During the drying process, the variations in the contents of different volatile compounds are inconsistent with the decrease in moisture content [8]. The variation in volatile compounds according to moisture content in shrimp during drying has been rarely studied.

Amino acids, the most abundant component in fresh shrimp apart from water, are an important precursor substance affecting the aroma of shrimp after drying. To explore their effects on volatile compounds, several models have been constructed. For example, Yu et al. [9] and Adams et al. [10] investigated the formation of pyrazines using the Maillard reaction model between L-glutamic acid and multiple amino acids. Although many related models have been studied, their results may be unrealistic due to the complexity of the food matrix. Therefore, to study the role of amino acids in the formation of volatile compounds, single amino acids have been added to a food matrix [11]. However, there are relatively few reports investigating the addition of amino acids to shrimp during drying and their effect on volatile compounds. In addition, the amino acids of different species have different properties, resulting in the formation of different volatile compounds [12]. The contributions of these compounds to the aroma characteristics of shrimp are inconsistent. For example, 2-methylbutyraldehyde is produced by isoleucine and imparts a characteristic nutty odor [13]. In comparison, 3-(methylthio) propionaldehyde is obtained by sulfur-containing amino acids, contributing to an odor resembling cooked meat and onion [14]. Therefore, it is necessary to screen the amino acids that produce volatile compounds in shrimp, and then verify the role of amino acids in the formation of volatile components via addition assay. Screening of amino acids related to the production of volatile compounds during shrimp drying has not yet been reported.

In this study, GC-IMS and GC-MS were used to analyze the variations in the volatile components of shrimp during drying. An amino acid automatic analyzer was employed to measure the changes in amino acid content during processing. Correlations between amino acids and important volatile compounds were analyzed in terms of the Pearson correlation coefficient to screen out the amino acids involved in their production. The screened amino acids were added to raw shrimp, which were then dried to reveal their effect on volatile compound formation.

## 2. Materials and Methods

### 2.1. Sample Preparation

Fresh white shrimp (*Penaeus vannamei*) with an average weight of 20.0 g were purchased at a local market in Zhanjiang City, Guangdong Province, China in May 2022. The raw shrimp were first processed evenly by a blender, after which a 1 mm thick coating of shrimp was applied to glass plates. The glass plates were placed into an electric thermostat blast oven (Eyela NDO-710, Tokyo Rikakikai Co., Ltd., Tokyo, Japan) for drying. The internal temperature of the oven was maintained at 85 °C. A total of five samples with different moisture contents were obtained. These five samples were labeled as follows: raw shrimp (RS), 45% shrimp (45%S), 30% shrimp (30%S), 15% shrimp (15%S), and 5% shrimp (5%S), respectively. All dried samples were packed in airtight containers and stored at −18 °C until use.

### 2.2. HS-GC-IMS Analysis

According to the slightly modified method of Li et al. [15], volatile compounds from shrimp were identified by GC-IMS (FlavourSpec^®^, G.A.S. Dortmund, Germany). The shrimp sample (0.5 g) was loaded into a 20 mL headspace bottle and incubated at 60 °C for 15 min. Then, 500 μL of gas from the headspace bottle was injected into the injection port (80 °C) by means of a heated syringe. The gas was pre-separated by a gas chromatographic column (MXT-5, 15 m × 0.53 mm × 1 μm, Restek, Bellefonte, PA, USA) at 60 °C. The pre-separated compounds were ionized and further separated in the IMS ionization chamber (45 °C). The flow rate of the carrier gas (nitrogen, 99.99% purity) was as follows: firstly 5 mL/min for 10 min, after which it increases linearly to 150 mL/min within 5 min. The flow rate of the drift gas (nitrogen, 99.99% purity) was maintained at 150 mL/min and the drift tube was 98.0 mm long. The retention index (RI) of volatile compounds was evaluated using C4-C9 n-ketones (Sinopharm Chemical Reagent Beijing Co., Ltd., Beijing, China) as an external reference under the same chromatographic conditions. Volatile compounds were identified based on the RI and the drift times (DT) of standard compounds compared to the GC-IMS database on G.A.S. Difference comparison plot (a direct comparison with among samples) and Gallery plot were constructed by using the VOCal software.

### 2.3. HS-SPME-GC-MS Analysis

The extraction and analysis of volatile compounds were carried out following the method of Zhang et al. [6] with slight modifications. A 2.00 g sample was accurately weighed into a 40 mL headspace bottle, to which 2 μL internal standard solution (methyl nonanoate, 0.21875 g/L in methanol) was added. The headspace bottle was placed into a 60 °C water bath for 10 min to achieve a balance of gas in the headspace. The SPME needle (DVB/CAR/PDMS, 1 cm, 50/30 μm; Supelco, Bellefonte, PA, USA) was exposed to the headspace and extracted for 35 min.

After extraction, the SPME needle was inserted into the GC injector of a TQ8050NX gas chromatograph–mass spectrometer system (Shimadzu, Kyoto, Japan) and desorbed at 240 °C for 5 min. Volatile compounds were separated using an InertCap^®^ Pure-WAX capillary column (30 m × 0.25 mm, 0.25 µm film thickness; Shimadzu, Japan). The carrier gas was helium (99.999% purity) at a flow rate of 1.0 mL/min. The column temperature procedure was as follows: maintain a temperature of 40 °C for 3 min; ramp up to 100 °C at 4 °C/min; hold for 2 min; ramp up to 230 °C at 8 °C/min; maintain for 5 min. The temperature of quadrupole and ion source was 230 °C. The ion source was operated in electron impact (EI) mode at 70 eV. Full scan mode was adopted across the *m*/*z* range of 33–550.

### 2.4. Identification and Quantitative Analysis of Volatile Compounds

Each volatile compound was identified by matching mass spectra to the database (NIST05 and Wiley07) [16]. All AACs were identified by comparison to known standards.

The concentration of each volatile compound was determined by the ratio of the peak area to the internal standard (IS) peak area under comparable GC–MS conditions. The concentration of each volatile compound was calculated using the following formulae:

(1) $$C_{i}=\frac{A_{i}\times C_{S}}{A_{S}}\times f_{i}$$

C_i_: the content of the volatile compound detected in the sample; A_i_: the compound peak area; C_s_: the IS concentration (ng/g); A_s_: the IS peak area; *f_i_*: the calibration factors (all assumed as 1.0).

### 2.5. Odor Activity Value (OAV)

OAV was calculated using the following formulae [17]:

(2) $$\mathrm{OAV}=\frac{C_{i}}{{\mathrm{OT}}_{i}}$$

C_i_: the content of the volatile compound detected in the sample; OT_i_: the sensory thresholds for odor in water.

### 2.6. Determination of Amino Acids

The qualitative and quantitative amino acids of shrimp were analyzed by an automatic amino acid analyzer determination, according to the Chinese National Food Safety Standard GB 5009.124-2016. Each sample was accurately weighed 0.2000 g into the hydrolysis tube, adding 15 mL of 6 M hydrochloric acid (Beijing Chemical Works, Beijing, China). Three drops of phenol were aspirated into the tube. The tube was placed at 110 °C and heated for 22 h after 10 min of nitrogen blowing. The hydrolysate was filtered and fixed with distilled water to 100 mL. 1 mL of diluent was aspirated into a 25 mL tube and dried under reduced pressure conditions at 40–50 °C to obtain the residue. The residue was dissolved in 2.0 mL sodium citrate (pH 2.2) and filtered through a membrane with a pore size of 0.22 µm (Anpel Laboratory Technologies Inc., Shanghai, China). Amino acids were separated using an automatic amino acid analyzer (L-8900, Hitachi, Tokyo, Japan). The content of amino acids was expressed on a dry weight basis.

### 2.7. Addition Assay

Raw shrimp samples were spiked with different single amino acids. The amount of each amino acid added was based on the amino acid content of the raw shrimp at a 1:1 ratio. The raw shrimp samples with added amino acids were frozen in liquid nitrogen and mixed evenly using a blender. The raw shrimp samples were dried as described in Section 2.1, and finally samples with 5% moisture content were obtained. These samples were, respectively, named the Phe group (PheG), the Leu group (LeuG), the Ile group (IleG), the Lys group (LysG), the His group (HisG), and the Arg group (ArgG).

### 2.8. Statistical Analysis

All experiments were repeated in triplicate and data were expressed as mean ± standard deviation (SD). One-way analysis of variance (ANOVA), *t*-test, and Pearson correlation coefficient analysis were performed using SPSS 23 software (IBM, Armonk, NY, USA) to analyze significant differences (*p* < 0.05, two-tailed).

## 3. Results and Discussion

### 3.1. GC-IMS Analysis

**Figure 1:**
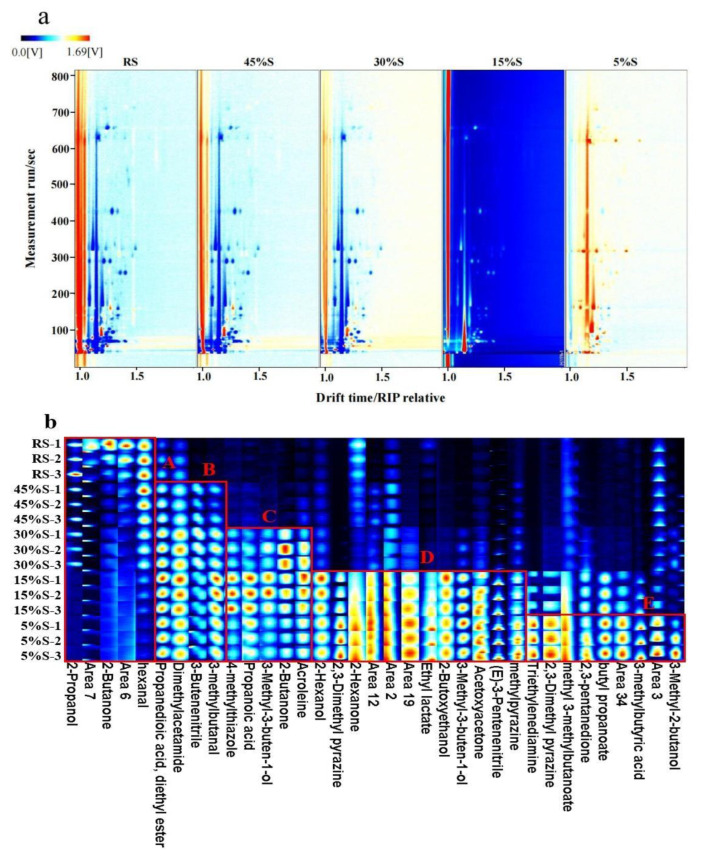
Difference comparison plots (**a**) and gallery plot (**b**) of volatile components of shrimp samples with different moisture contents (each column represents a compound, each row represents a sample, and the signal peak color represents the substance concentration, the darker the color, the greater the concentration).

The HS-GC-IMS method was used to obtain global IMS information of the shrimp during the drying process to identify the volatile substances and analyze their variation regularity. Figure 1a shows a two-dimensional GC-IMS analysis spectrum of the shrimp in the reporter plot mode. The vertical coordinate indicates the GC retention time (Rt), the horizontal coordinate indicates the ion drift time (Dt), and the vertical line at 1.0 of the horizontal coordinates indicates the reactive ion peak (RIP). Each point on the right of the RIP represents a volatile substance. According to the results of GC-IMS and GC-MS, during the process of reducing the moisture content from 30% to 15%, the types and contents of volatile substances in shrimp changed significantly. Therefore, the spectrum of the 15%S sample was selected as a reference in the graph, and the spectrums of the other samples were deducted from the reference. After deduction, white indicates that the concentration of the volatile substances was the same, a red dot indicates that the concentration was higher than that of the reference, and a blue dot indicates that the concentration was lower than that of the reference [18]. In the right-hand area of the RIP, compared to 15%S, the plots of the higher moisture content samples (RS, 45%S, 30%S) were predominantly blue, while the plot of the lower moisture content sample (5%S) was predominantly red, indicating that volatile substances increased in concentration or new volatile substances were generated as the moisture content decreased during the drying process. The most obvious changes occurred in the moisture content range of 30% to 5%.

In Figure 1b, a total of 28 types of volatile compounds were identified in the five samples, including 3 pyrazines, 3 aldehydes, 5 ketones, 6 alcohols, 2 acids, 4 esters, and 5 unidentified substances. The data in the GC-IMS database were limited, making it impossible to characterize all substances [15]. It could be seen that the peak intensities and peak numbers of the volatile compounds in the five samples were not similar, which indicated that the overall volatile substances of the five samples were not basically the same. During the drying process, some peak colors changed from red-orange to blue, and vice versa, indicating that some volatile compounds disappeared and some new compounds were generated, respectively. Overall, with decreases in shrimp moisture content, the volatile components increased in type and content. To make it easier to distinguish the differences in volatile compounds in the samples, Figure 1b was divided into five parts, labelled with five matrices (A, B, C, D, E), according to the change in volatile compound content during the drying process. Matrix A indicated that several compounds were detected in raw shrimp, including 2-propanol, 2-butanone, and hexanal, but they disappeared gradually as the shrimp dried. Some pyrazines were detected in matrix D, including 2,3-dimethyl pyrazine and methyl pyrazine. The results were also found in GC-MS, indicating that pyrazines were gradually produced in the later stages of drying. The pyrazines were mainly produced via Maillard reaction, mainly in their final stage, which has a faster reaction rate and more products at lower moisture levels [19]. The GC-IMS results indicated a significant change in the type and content of volatile compounds in the shrimp during the drying process.

### 3.2. HS-SPME–GC–MS Analysis

**Figure 2:**
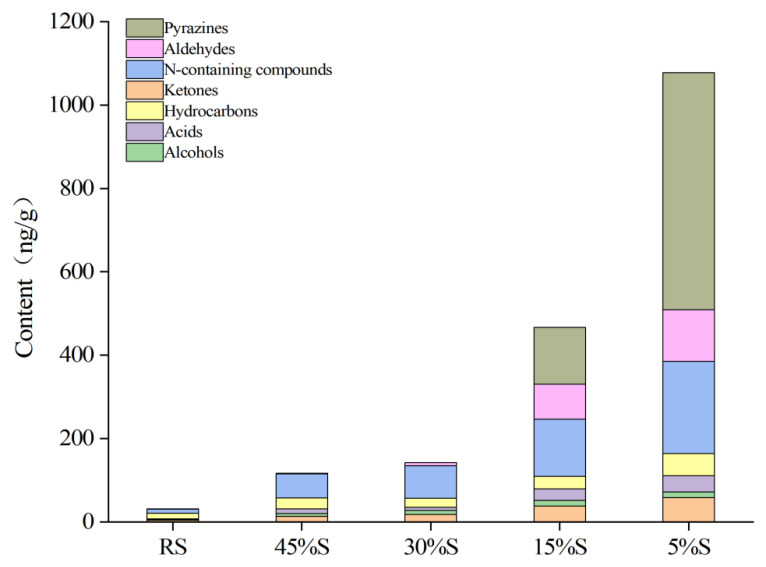
Changes of volatile components in shrimp samples with different moisture contents.

Volatile components in the shrimp samples were analyzed by HS-SPME–GC–MS. A total of 73 volatile compounds were identified, which were sorted into seven classes, namely, pyrazines (20), aldehydes (7), ketones (12), alcohols (6), acids (12), N-containing compounds (4), and hydrocarbons (12), as shown in Table S1. Most of these volatile compounds have been previously reported in several shrimp species [3,6]. The composition of volatile compounds varied at different moisture contents. In the early stage of drying, only 35 volatile compounds were detected. Samples with decreasing moisture content had an increased variety of volatile components. The number of volatile compounds increased to 68 species when the sample’s moisture content reached 5%. Similar changes were observed in the content of volatile compounds (Figure 2). In the early stage of drying, the contents of volatile components of samples did not change significantly. A decrease in moisture content from 30% to 5% resulted in a significant increase in volatile components content, from 143.31 ± 17.10 ng/g to 1103.35 ± 96.50 ng/g. GC-IMS and GC-MS analyses confirmed a drastic change in volatile compound composition and content in shrimp samples with moisture contents from 30% to 5%.

In Figure 2, pyrazines, aldehydes, and N-containing compounds were abundantly produced in the 30%S and 5%S samples. These compounds have been widely reported in dried shrimp products [6], and are mainly derived from Maillard reaction products, lipid degradation products, and products generated through interactions. There were significant differences in the volatile flavor profiles of the dried shrimp samples from 30% to 5% moisture content due to the production and increased content of these compounds.

Pyrazines are low-threshold and high-content compounds in dried shrimp products. They have a pleasant roasted flavor and are considered an important class of roasted and cooked-meat-like odorants. Pyrazines resulting from the Maillard reaction—which involves free amino acids, peptides, proteins, and reducing sugars—were formed by a reaction between heat-induced carbohydrate degradation products and Strecker products of amino acids or ammonia [20]. In this study, pyrazines were identified in both the 15%S and 5%S samples, with the latter having a higher content and variety of pyrazine compounds. In 5%S, a total of 20 pyrazines were identified with a total content of 568.84 ± 36.5 ng/g, accounting for 52.79% of the total volatile compounds content. Therefore, the highest content of volatile compounds in shrimp were pyrazines. The increase in pyrazine content in the late drying stage was due to the decrease in water activity, which enhances the Maillard reaction, resulting in the production of large amounts of Maillard reaction products [19]. Therefore, in subsequent studies, the determination of amino acids involved in the Maillard reaction, and the production of pyrazines should focus on the amino acids with contents that decrease as the moisture content decreases from 30% to 5%.

Aldehydes, which have a low odor threshold value, are important aroma compounds in dried shrimp products and exert a strong influence on their aroma. During the drying process, the aldehyde content varies similarly to that of pyrazines. Aldehydes were shown to increase in the 15%S significantly and 5%S samples compared to the other samples. In 5%S, 2-methylbutanal, 3-methylbutanal, and benzaldehyde were the three aldehydes with the highest contents. In extant research, these three aldehydes are considered key aroma compounds in dried shrimp products [6]. They are mainly produced by the Strecker degradation of amino acids, and their corresponding precursors are isoleucine, leucine, and phenylalanine, respectively. Therefore, subsequent studies looking to identify the amino acids that participate in the Maillard reaction and give rise to important aroma compounds should focus on Leu, Ile and Phe.

Trimethylamine, which was found in all samples, was one of the measured volatile compounds with the most significant changes in contents. The increase in trimethylamine during heating is due to thermal decomposition of choline, betaine, or trimethylamine oxide [21].

In addition to the compounds discussed above, other products of the Maillard reaction are shown in Table S1 and include benzyl alcohol, 5-methyl-2-furanmethanol, 2,3-dihydro-3,5-dihydroxy-6-methyl-4H-pyran-4-one, and 3-methyl-butanoic acid. In conclusion, several Maillard reaction products were found in the volatile compounds detected in the shrimp (15%S and 5%S). This indicates that the Maillard reaction plays an important role in the aroma of dried shrimp products. The amino acids involved in the Maillard reaction are among the main precursors for the production of volatile compounds in shrimps.

### 3.3. Aroma-Active Components in Different Shrimp Samples

The importance of aroma components in shrimp depends not only on the content of these compounds, but also on their OAV [22]. The OAV can be calculated according to the above concentrations and thresholds values of volatile compounds. Compounds with an OAV > 1 were identified as AACs. As shown in Table 1, 11 AACs (OAV > 1) were identified that contributed significantly to the overall aroma of shrimp.

At the initial stage of drying, the AAC species of RS and 45%S were mainly lipid oxidation degradation products. Their odor is mainly described as fishy and green, lacking roasted flavor. 1-Octen-3-ol has been identified as producing a mushroom-like odor and is an AAC that is found widely in seafood [23]. 1-Octanol imparts a characteristic sweet, citrusy-floral odor, which is detectable in both raw and boiled shrimp [5,24]. 2-Acetyl-1-pyrroline, which imparts a popcorn-like flavor, was first detected in 30%S and is considered to be an important flavor compound in dried shrimp products due to its low threshold [2].

The decrease in moisture content increased the Maillard reaction rate, causing the reaction products to accumulate and change the flavor profile of shrimp significantly (15%S and 5%S). Among the samples, the most significant changes were observed in the 5%S sample, with the most AACs species (10) and the highest OAV summation (1818.47). In 5%S, OAV summation of pyrazines was highest (1020.06), accounting for 56.09% of the total. Thus, pyrazines made the greatest contribution to the overall OAV of shrimp. Pyrazines such as 2-ethyl-5-methylpyrazine, 2-ethyl-3,6-dimethylpyrazine, and 2,5-dimethylpyrazine mainly impart a pleasant roasted nutty and roasted meat flavor, and significantly contribute to the overall aroma of dried shrimp products [3].

With the extension of drying time, the content of trimethylamine gradually increased, and it became an AAC in shrimp. The results of this study were consistent with the report of Zhang et al. [6]. Generally, the flavor of trimethylamine was described as fishy and grassy, but the flavor of shrimp in the later stage of drying was described as having a roasted taste. Whether the fishy smell of trimethylamine was covered by other ACCs or synergized with other compounds to produce a roasted flavor is worth exploring.

### 3.4. Analysis of Amino Acid and Correlation

Amino acids play an important role in the formation of food flavor. They can participate not only in thermal degradation reactions, but also in Maillard reactions. These reactions produce a variety of volatile compounds that contribute to the complexity of food flavors [25]. The changes in amino acid content in shrimp during drying are shown in Table 2. In the drying process, the total content of amino acids decreased significantly, with the extent of decrease differing by amino acid type (except proline). According to the analysis of the volatile components of shrimp, shrimp with a moisture content of 30% and 5% contained the most Maillard reaction products. At this stage, the four amino acids with the largest reductions in amino acid contents were Glu (2.47 g/100 g), Lys (1.88 g/100 g), Asp (1.34 g/100 g), and Arg (1.20 g/100 g).

**Figure 3:**
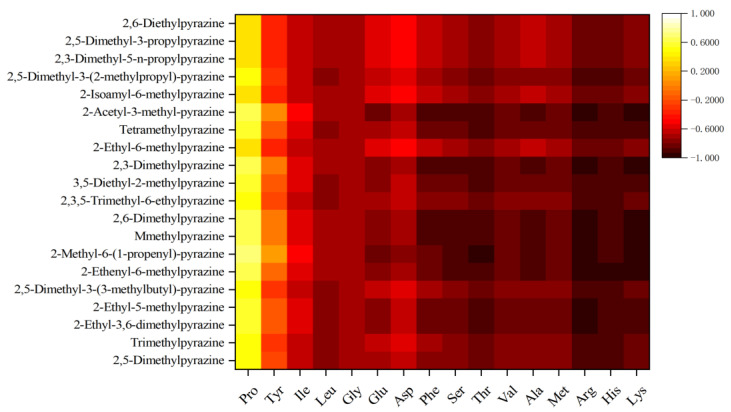
Pearson correlation coefficient diagram of pyrazines and amino acids.

A significant change in pyrazine was observed during shrimp drying according to the results in Section 3.1 and Section 3.2. Pyrazine, which contributes a pleasant roasted nutty and roasted meat flavor, had the most significant changes in content and species with the highest OAV percentage, while its precursors were mainly amino acids. Therefore, a Pearson correlation coefficient was determined for the relationship between the content of amino acids and pyrazines in shrimp [26]. As demonstrated in Figure 3, 14 amino acids (not Pro and Tyr) were negatively correlated with all pyrazines, and the Pearson correlation coefficients were different. Compared to other amino acids, the basic amino acids (Arg, Lys, and His) had high negative correlations with all pyrazines, with Pearson’s correlation coefficients range from −0.739 to −0.99. Sohn et al. [27] found that some amino acids such as Lys and His contain more than one nitrogen atom, which contributes to the formation of nitrogen-containing heterocycles such as pyrazines, pyridines and pyrroles. The Maillard reaction has shown to be strongly dependent on the reaction’s pH, with a weakly alkaline environment (pH < 10) producing a more favorable reaction [28]. Basic amino acids (Arg, Lys, His) can provide such an environment. Laroque et al. [29] also found that Lys is Hig-reactive in the Maillard reaction and contributes to its global reactivity. According to the analysis of amino acid content changes and Pearson’s correlation, basic amino acids (Arg, Lys, His) may be aroma precursors that are the main participants in the reaction and produce pyrazine aroma compounds. Therefore, these three amino acids were selected for the addition assay study.

The results of the subsequent addition assay revealed that the contents of aldehydes in shrimp were reduced after the addition of basic amino acids. So, the amino acids (Ile, Leu, and Phe) corresponding to the aldehydes (2-Methylbutanal, 3-Methylbutanal, and Benzaldehyde) of AACs in shrimp were selected for additional tests. Their corresponding relationship have been demonstrated previously [30,31]. Thus, Ile, Leu, and Phe were determined to be the important amino acids in raw shrimp that participate in the Maillard reaction and give rise to aroma-active components.

### 3.5. Analysis of the Addition Assay Results

**Figure 4:**
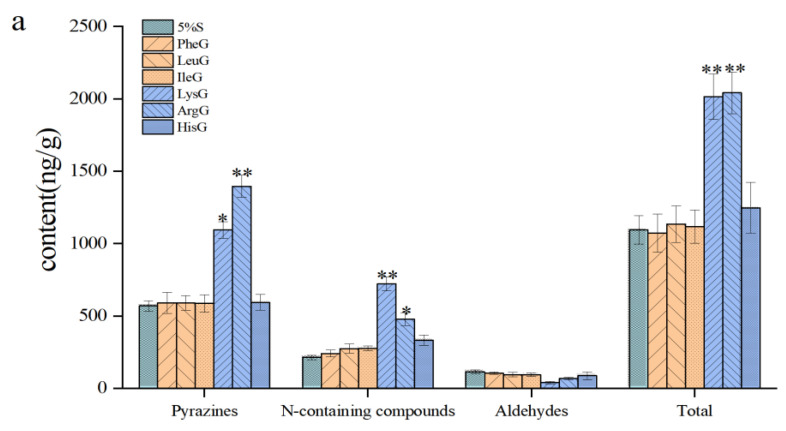

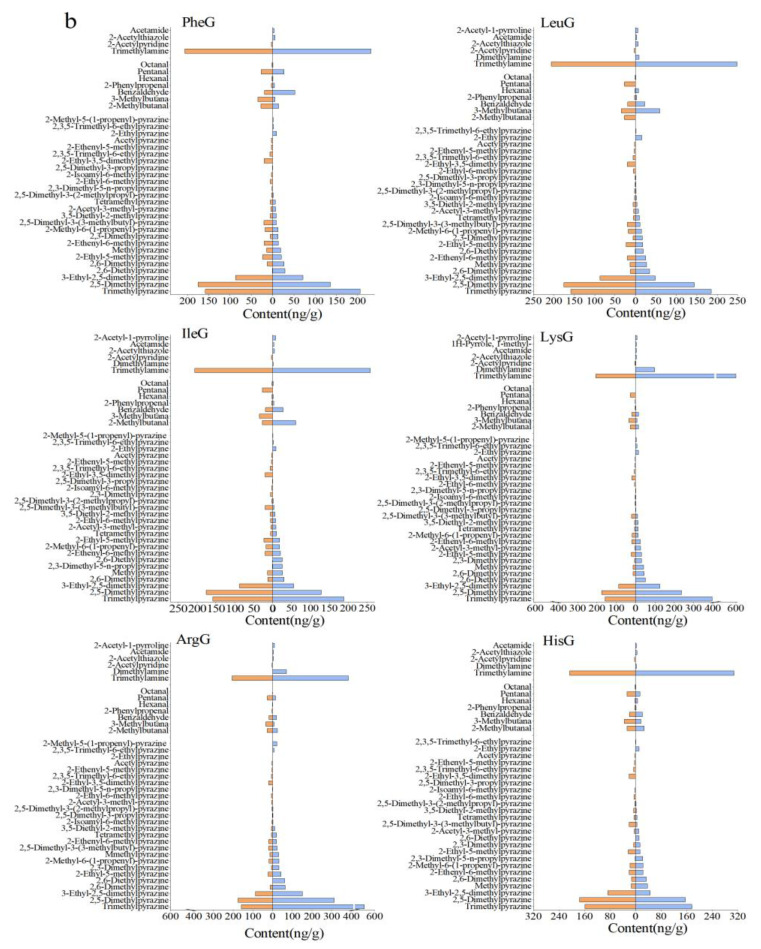
Contents of volatile components of different classes in shrimp before and after adding amino acids (**a**) (** indicates significant difference compared to 5%S (*p* ≤ 0.01); * indicates significant difference compared to 5%S (*p* ≤ 0.05)) and comparison of different pyrazines, N-containing compounds and aldehydes in shrimp before and after adding amino acids (**b**) (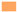 The volatile compounds in 5%S; 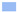 The volatile compounds in shrimps after adding amino acids).

To verify the potential role of the above amino acids in the formation of the unique flavor of shrimp, the raw shrimp with added amino acids were dried in a way that simulated the drying conditions of the shrimp. The volatile components of the shrimp before and after the addition of amino acids were then compared [32,33]. Figure 4a and Table S2 show the contents of pyrazines, N-containing compounds, aldehydes, and total volatile components in the shrimp before and after adding amino acids. Figure 4b shows the types and contents of pyrazines, N-containing compounds, and aldehydes in shrimps at 5%S and after the addition of amino acids.

As shown in Figure 4a, LysG, hisG, and ArgG were the three groups observed to increase as proportions of the total volatile compounds. Among these, LysG and ArgG increased significantly compared to 5%S, with 1.84 and 1.86-fold increases, respectively. Among these two groups, pyrazines and N-containing compounds were the two classes of volatile compounds with the greatest increase in content. This phenomenon confirms that these two amino acids contributed to the formation of nitrogen-containing heterocycles. As shown in Figure 4b, a total of 21 pyrazines were detected in LysG and ArgG, with contents of 1094.31 ± 57.47 ng/g and 1396.76 ± 77.36 ng/g, respectively, equating to 1.84- and 2.35-times the contents found in 5%S. In ArgG, the contents of ACCs (2,5-Dimethylpyrazine, 2-Ethyl-5-methylpyrazine, 2-Ethyl-3,6-dimethyl pyrazine) increased substantially compared to those in 5%S, at 312.83 ± 5.19, 43.65 ± 1.06, and 151.87 ± 20.77 ng/g, respectively. The changes in the above ACCs of LysG were similar to those of ArgG, except for a less significant change in their contents. According to the above analysis, Maillard reactions involving Lys and Arg can play an important role in generating the pleasant roasted nutty flavor compounds of shrimp. However, the types and contents of aldehydes were substantially reduced, decreasing by 65.52% and 34.23%, respectively, compared to 5%S. The addition of amino acids promotes further reactions of carbonyl compounds [34]. In addition, the addition of basic amino acids increases the trimethylamine content and causes dimethylamine to appear.

In LeuG, IleG and PheG, the contents of the corresponding Strecker aldehydes (3-Methylbutanal, 2-Methylbutanal, and Benzaldehyde) increased to 59.59 ± 3.38 ng/g, 60.76 ± 7.55 ng/g and 52.20 ± 3.07 ng/g, respectively, which were 1.71-, 2.23- and 1.79-times those of 5%S. As such, these aldehydes increased the AOV values of the corresponding AACs in shrimp. Although the three groups mentioned above had no inhibitory effect on the production of pyrazines, the AACs of pyrazines were lower compared to those of 5%S. Therefore, to maintain the flavor of the shrimp, these three amino acids were added along with either Lys or Arg.

## 4. Conclusions

The AACs of shrimp changed significantly throughout the drying process. The moisture content and the type of amino acids were important factors influencing the formation of AACs. As the moisture content of the shrimp decreased, both the content of volatile compounds and their types increased. Amino acids were involved in the production of volatile compounds in shrimp during drying. Lys and Arg were precursors of pyrazines during shrimp drying. These results form a scientific basis for clarifying the mechanism of aroma formation in shrimp.

## Figures and Tables

**Table 1:** Odor thresholds and aroma-active compounds in shrimp.

Compound	Threshold	OAV				
		5%S	15%S	30%S	45%S	RS
2,5-Dimethylpyrazine	0.8 c	219.26	41.89	ND	ND	ND
2-Ethyl-3,6-dimethylpyrazine	0.4 b	217.80	70.65	ND	ND	ND
2-Ethyl-5-methylpyrazine	0.04 b	583.00	189.75	ND	ND	ND
2-Methylbutanal	1.5 a	18.17	14.15	2.67	ND	ND
3-Methylbutanal	0.5 a	69.84	52.20	ND	ND	ND
Benzaldehyde	28 c	1.04	0.68	0.10	0.07	0.01
Octanal	0.587 b	2.04	1.21	ND	ND	ND
1-Octen-3-ol	0.016 a	472.50	583.75	125.00	65.00	13.75
1-Octanol	1 b	ND	ND	1.71	1.77	1.22
Trimethylamine	2.4 b	85.96	56.29	32.71	24.17	4.22
2-Acetyl-1-pyrroline	0.053 a	148.86	82.07	28.11	ND	ND
Total		1818.47	1092.64	190.31	90.01	18.19

Threshold refers to the following literature: a [2], b [5], c [6], ND = not determined.

**Table 2:** Amino acid contents of shrimp (g/100 g sample, dry weight basis).

Amino Acid	RS	45%S	30%S	15%S	5%S
Ala	4.55 ± 0.01 a	4.47 ± 0.01 a	3.95 ± 0.06 b	3.45 ± 0.07 c	3.24 ± 0.03 c
Arg	6.00 ± 0.01 a	6.03 ± 0.02 a	5.62 ± 0.05 a	5.00 ± 0.08 b	4.42 ± 0.06 c
Asp	6.86 ± 0.03 a	4.37 ± 0.02 b	3.18 ± 0.08 c	2.11 ± 0.02 d	1.84 ± 0.06 d
Glu	10.86 ± 0.01 a	8.54 ± 0.01 b	6.53 ± 0.03 c	4.76 ± 0.02 d	4.06 ± 0.04 e
Gly	5.09 ± 0.01 a	4.95 ± 0.02 a	4.93 ± 0.01 a	4.93 ± 0.01 a	4.84 ± 0.08 a
His	1.41 ± 0.02 a	1.55 ± 0.04 a	1.47 ± 0.05 a	1.39 ± 0.00 a	1.24 ± 0.02 b
Ile	2.64 ± 0.01 a	2.46 ± 0.01 b	2.32 ± 0.00 c	2.51 ± 0.05 b	2.26 ± 0.02 c
Leu	5.86 ± 0.01 a	5.15 ± 0.02 b	5.18 ± 0.02 b	5.23 ± 0.09 b	4.67 ± 0.08 c
Lys	5.50 ± 0.05 a	4.99 ± 0.01 b	4.81 ± 0.01 b	3.53 ± 0.01 c	2.93 ± 0.06 d
Met	0.41 ± 0.00 a	0.35 ± 0.00 b	0.33 ± 0.03 bc	0.32 ± 0.01 bc	0.27 ± 0.00 c
Phe	2.86 ± 0.01 a	2.86 ± 0.01 a	2.85 ± 0.01 a	2.76 ± 0.01 a	2.76 ± 0.05 a
Pro	3.05 ± 0.01 c	5.53 ± 0.02 b	5.72 ± 0.02 b	6.99 ± 0.06 a	6.66 ± 0.01 a
Ser	2.64 ± 0.04 a	2.68 ± 0.00 a	2.34 ± 0.01 b	2.10 ± 0.01 c	1.96 ± 0.01 d
Thr	2.55 ± 0.00 a	2.67 ± 0.06 a	2.35 ± 0.07 b	2.07 ± 0.02 c	1.85 ± 0.04 d
Tyr	2.73 ± 0.01 a	2.50 ± 0.01 a	2.61 ± 0.09 a	2.81 ± 0.02 a	2.55 ± 0.03 a
Val	3.14 ± 0.03 a	2.70 ± 0.09 b	2.63 ± 0.01 b	2.46 ± 0.03 c	2.16 ± 0.03 c
Total content	66.15 ± 0.26 a	61.80 ± 0.35 b	57.12 ± 0.55 c	52.55 ± 0.51 d	48.06 ± 0.62 e

Different letters indicated that the significant differences (*p* < 0.05) in the same row.

## Data Availability

The datasets generated for this study are available on request to the corresponding author.

## Supplementary Materials

The following supporting information can be downloaded at: https://www.mdpi.com/article/10.3390/foods11203264/s1; Table S1: Volatile compounds identified in shrimp of different moisture contents; Table S2: Volatile compounds of dried shrimp from the addition assays.
