# Supplementary material for: The Role of Amino Acids in the Formation of Aroma-Active Compounds during Shrimp Hot Air Drying by GC-MS and GC-IMS
Source: Foods. 2022 Oct 19;11(20):3264. doi: 10.3390/foods11203264 (PMC9601334; doi:10.3390/foods11203264)
Supplement: Supplementary file 1 [file foods-11-03264-s001.zip › foods-1926504-supplementary.pdf]

foods

Table S1. Volatile compounds identified in shrimp of different moisture contents.

Compound（ng/g）	5%S	15%S	30%S	45%S	RS
Pyrazines (20)					
2,5-Dimethylpyrazine	175.41±8.55	33.51±2.50	ND	ND	ND
Trimethyl pyrazine	158.27±14.47	22.87±5.72	ND	ND	ND
2-Ethyl-3,6-dimethylpyrazine	87.12±1.79	28.26±1.49	ND	ND	ND
2-Ethyl-5-methylpyrazine	23.32±0.48	7.59±0.50	ND	ND	ND
2,5-Dimethyl-3-(3-methylbutyl)-pyrazine	20.23±1.47	3.04±0.17	ND	ND	ND
2-Ethenyl-6-methylpyrazine	20.06±1.82	8.55±0.56	ND	ND	ND
2-Methyl-6-(1-propenyl)-pyrazine	17.6±1.32	11.01±0.52	ND	ND	ND
Methyl pyrazine	13.72±1.59	6.16±0.81	ND	ND	ND
2,6-Dimethylpyrazine	12.72±1.19	5.75±0.24	ND	ND	ND
2,3,5-Trimethyl-6-ethylpyrazine	6.29±0.54	1.30±0.00	ND	ND	ND
3,5-Diethyl-2-methylpyrazine	5.87±0.27	1.80±0.45	ND	ND	ND
2,3-Dimethylpyrazine	5.76±0.28	2.69±0.31	ND	ND	ND
2-Ethyl-6-methylpyrazine	5.42±0.47	ND	ND	ND	ND
Tetramethyl pyrazine	5.32±0.93	1.60±0.27	ND	ND	ND
2-Acetyl-3-methyl-pyrazine	4.32±0.58	2.29±0.16	ND	ND	ND
2-Isoamyl-6-methylpyrazine	2.48±0.35	ND	ND	ND	ND
2,5-Dimethyl-3-(2-methylpropyl)-pyrazine	2.22±0.08	0.33±0.01	ND	ND	ND
2,3-Dimethyl-5-n-propylpyrazine	1.01±0.20	ND	ND	ND	ND
2,5-Dimethyl-3-propylpyrazine	0.93±0.10	ND	ND	ND	ND
2,6-Diethylpyrazine	0.77±0.02	ND	ND	ND	ND
Subtotal	568.84±36.5	136.75±13.71	0±0	0±0	0±0
Aldehydes （7）					
2-Methylbutanal	27.26±3.23	21.23±1.32	4.01±2.01	ND	ND
3-Methylbutana	34.92±5.24	26.10±0.00	ND	ND	ND
Benzaldehyde	29.10±0.66	19.07±0.90	2.71±0.10	1.99±0.06	0.14±0.00
Octanal	1.20±0.27	0.71±0.05	ND	ND	ND
Hexanal	1.79±0.43	1.72±0.88	0.31±0.00	ND	ND
Pentanal	27.2±0.94	20.20±0.49	ND	ND	ND
2-Phenylpropenal	2.58±0.74	1.03±0.33	0.23±0.02	ND	ND
Subtotal	124.05±11.51	90.06±3.97	7.26±2.13	1.99±0.06	0.14±0
Ketones（12）					
2,3-Dihydro-3,5-dihydroxy-6-methyl-4H-pyran-4-one	13.27±1.64	4.21±0.57	ND	ND	ND
2,5-Dimethylfuran-3,4(2H,5H)-dione	0.96±0.03	0.23±0.00	ND	ND	ND
2-Pyrrolidinone	2.71±0.19	1.13±0.13	0.54±0.13	0.44±0.14	ND
2-Heptanone	5.67±1.19	6.06±0.88	ND	ND	ND
2-Decanone	7.05±0.32	4.71±0.22	ND	ND	ND
2-Piperidinone	4.19±0.15	1.60±0.41	0.22±0.05	0.28±0.00	ND
2-Nonanone	9.76±2.1	13.94±0.43	12.45±1.06	8.34±1.27	2.38±1.09
2-Undecanone	1.60±0.00	1.12±0.14	0.45±0.00	0.52±0.13	ND
2-Octanone	4.96±0.27	3.57±0.13	0.91±0.01	0.5±0.00	0.12±0.05
Trans-geranyl acetone	1.10±0.00	1.59±0.78	0.56±0.11	0.83±0.19	0.37±0.10.
6-Methyl-hept-5-en-2-one	ND	ND	0.64±0.16	0.53±0.01	0.47±0.18
5-Methyl-2-hexanone	6.78±0.00	ND	1.89±0.13	1.75±0.00	1.03±0.00
Subtotal	58.05±5.89	38.16±3.69	17.66±1.65	13.19±1.74	4.37±1.32
Alcohols（6）					
1-octen-3-ol	7.56±0.56	9.34±0.00	2.00±0.09	1.04±0.19	0.22±0.07
Benzyl alcohol	4.18±0.37	0.81±0.10	0.28±0.01	ND	ND
1-Pentanol	ND	2.62±0.42	3.65±0	2.74±0.49	0.57±0.00
2-Ethyl-hexanol	1.63±0.00	ND	1.89±0.01	1.93±0.53	0.60±0.26
1-Octanol	ND	ND	1.71±1.02	1.77±0.03	1.22±0.38
Tridecanol	0.52±0.06	0.40±0.07	0.32±0.09	ND	0.06±0.08
Subtotal	13.89±0.99	13.17±0.59	9.85±1.22	7.48±1.24	2.67±0.79
Acids（12）					
Diethyl-acetic acid	4.05±0.00	4.76±1.88	ND	ND	ND
3-Methyl-butanoic acid	ND	ND	1.7±0.42	1.84±0.06	ND
3-Methyl-pentanoic acid	9.08±1.69	5.10±0.39	ND	ND	ND
Benzoic acid	1.07±0.17	0.82±0.05	0.41±0.10	ND	ND

Trans-2-decenoic acid	6.23±0.37	4.07±0.54	1.02±0.34	2.43±0.53	ND
(E)-3-decenoic acid,	5.56±0.37	4.78±1.28	1.34±0.46	2.44±0.80	ND
Decanoic acid	3.45±0.30	1.55±0.57	0.37±0.12	0.75±0.12	ND
Octanoic acid	1.98±0.09	1.17±0.20	0.73±0.00	0.9±0.19	0.08±0.02
Acetic acid	ND	4.99±0.02	0.87±0.07	0.86±0.28	0.21±0.05
Tetradecanoic acid	1.44±0.25	0.90±0.00	0.19±0.06	1.76±0.71	0.35±0.08
Pentadecanoic acid	0.53±0.08	ND	0.37±0.10	0.61±0.08	0.23±0.05
Hexadecanoic acid	5.87±0.19	2.87±0.38	1.00±0.09	5.59±0.82	1.59±0.51
Subtotal	39.26±3.51	31.01±5.31	8.00±1.76	17.18±3.59	2.46±0.71
N-containing compounds（4）					
Trimethylamide	206.3±15.36	135.1±0.67	78.51±0.53	58.01±7.37	10.12±4.74
1-(1H-Pyrrol-2-yl)-ethanone	3.58±0.26	0.84±0.81	ND	ND	ND
1-(2-Pyridinyl)-ethanone	2.86±0.38	1.76±0.03	ND	ND	ND
2-Acetyl-1-pyrroline	7.89±0.56	4.35±0.36	1.49±0.07	ND	ND
Subtotal	220.63±16.56	142.05±1.87	80.00±0.60	58.01±7.37	10.12±4.74
Hydrocarbons (12)					
Tridecane	8.27±2.08	5.00±2.26	2.80±1.22	4.65±2.02	1.11±0.18
Undecane	8.95±3.46	2.15±0.95	2.16±0.86	1.53±0.61	1.87±0.42
Pentadecane	0.51±0.13	0.53±0.19	2.04±1.63	1.06±0.61	0.31±0.00
Tetradecane	1.44±0.29	1.28±0.44	0.79±0.15	1.09±0.31	0.57±0.13
Heptadecane	0.98±0.15	0.88±0.08	ND	1.32±0.36	ND
Dodecane	23.23±11.84	8.72±3.98	7.68±3.53	10.99±3.72	4.84±0.85
Decane	4.95±0.54	2.82±0.60	0.62±0.09	0.53±0.06	1.40±0.18
2,6,10,14-Tetramethyl-pentadecane	2.53±0.00	1.90±0.00	0.77±0.39	0.71±0.04	ND
2,6,10-Trimethyltridecane	2.01±0.57	1.49±0.15	0.73±0.22	0.43±0.05	ND
3,7-Dimethyl-decane	ND	1.97±0.25	1.79±1.23	1.01±0.28	1.76±0.28
2,6,11-Trimethyl-dodecane	ND	2.22±0.16	1.58±0.33	1.59±0.41	1.11±0.24
5-Methyl-tetradecane	ND	0.46±0.37	0.92±0.16	0.73±0.31	0.71±0.19
Subtotal	52.87±19.06	29.42±9.43	21.88±9.81	25.64±8.78	13.68±2.47
Total	1077.59±94.02	480.62±38.57	144.65±17.17	123.71±22.78	33.44±10.03

ND = not determined.

Table S2. Volatile compounds of dried shrimp from the addition assays.

**Compound(ng/g)**	**LysG**	**HisG**	**ArgG**	**PheG**	**LeuG**	**IleG**
Pyrazines (22)						
2,3,5-trimethyl-6-ethylpyrazine	6.91±0.58	1.27±0.82	8.11±1.24	2.28±0.69	1.53±0.02	2.34±0.32
Tetramethyl pyrazine,	14.00±1.16	4.85±2.90	20.29±4.12	6.56±0.24	9.62±1.66	10.44±1.83
Trimethyl pyrazine	397.21±4.81	176.39±15.54	547.68±14.65	206.01±22.89	185.50±11.10	188.05±16.8
2,3-Dimethyl-5-n-propylpyrazine	0.38±0.00	22.06±2.00	0.43±0.00	0.37±0.00	1.20±0.22	25.12±2.12
2,3-Dimethylpyrazine	30.89±6.31	13.72±1.00	32.2±5.12	12.59±0.06	16.64±4.00	ND
2,5-Dimethyl-3-(2-methylpropyl)-pyrazine	1.12±0.12	1.98±0.00	2.57±0.18	1.43±0.30	1.51±0.00	1.48±0.24
2,5-Dimethyl-3-(3-methylbutyl)-pyrazine	10.65±1.07	5.04±2.26	23.49±1.31	8.37±0.53	11.02±0.73	3.80±0.49
3,5-Diethyl-2-methylpyrazine	13.19±0.91	2.2±1.01	11.59±2.13	8.37±1.86	3.94±0.74	6.19±2.11
2,5-Dimethylpyrazine	237.97±8.06	155.71±7.00	312.83±5.19	135.37±18.28	144.07±12.59	128.57±19.22
2,6-Dimethylpyrazine	45.14±3.75	33.8±4.75	63.69±2.71	26.97±3.63	34.89±3.16	30.33±1.10
2,6-Diethylpyrazine	51.00±3.85	11.21±2.00	62.38±4.27	28.94±1.45	19.37±1.11	25.1±1.12
2-Methyl-5-(1-propenyl)-pyrazine	4.34±0.00	ND	0.82±0.00	1.55±0.18	ND	0.85±0.07
2-Methyl-6-(1-propenyl)-pyrazine	14.31±2.23	23.2±0.00	32.19±1.4	12.51±7.97	14.59±1.55	18.32±2.92
2-Isoamyl-6-methylpyrazine	0.86±0.00	ND	2.63±0.30	ND	2.29±0.38	ND
2-Ethenyl-6-methylpyrazine	24.80±1.59	23.43±2.35	20.71±2.39	13.13±2.88	25.31±0.80	20.51±1.75
2-Methylpyrazine	40.95±3.48	37.21±0	31.9±1.64	19.02±1.14	27.39±3.08	26.39±1.88
2-Ethyl-5-methylpyrazine	30.47±2.67	13.77±4.31	43.65±1.06	20.41±1.63	17.72±0.24	18.09±1.52
2-Ethyl-6-methylpyrazine	ND	ND	1.07±0.00	ND	ND	7.48±0.00
2-Ethylpyrazine	15.42±1.24	11.19±0	22.69±7.82	8.97±0.06	15.84±4.08	8.55±0.74
2-Acetyl-3-methylpyrazine	26.79±1.67	10.23±2.31	1.35±0.00	6.83±1.58	7.66±0.63	8.49±0.13
2,5-Dimethyl-3-propylpyrazine	1.40±0.63	ND	2.62±1.06	ND	0.63±0.00	ND
2-Ethyl-3,6-dimethylpyrazin	126.51±13.34	46.37±6.69	151.87±20.77	70.98±7.93	48.57±4.13	55.51±4.87
Subtotal	1094.31±57.47	593.63±54.94	1396.76±77.36	590.66±73.3	589.29±50.22	585.61±59.23
Aldehydes (7)						
2-Methylbutanal	15.44±5.09	27.04±9.41	25.11±2.00	13.99±1.23	ND	60.76±7.55
3-Methylbutanal	7.38±3.21	16.42±4.95	7.78±2.23	5.51±0.56	59.58±3.38	ND

Benzaldehyde	15.48±1.07	21.84±7.21	20.27±2.16	52.2±3.07	23.24±2.03	27.56±1.72
2-Phenylpropenal	1.02±0.13	1.42±0.93	ND	4.47±0.84	2.83±0.27	2.96±0.60
Hexanal	ND	6.25±1.27	ND	0.58±0.10	7.47±9.05	1.22±0.34
Pentanal	ND	13.69±2.48	15.32±3.13	26.79±2.20	ND	ND
Octanal	ND	0.82±0.00	ND	0.58±0.11	1.03±0.25	1.15±0.09
Subtotal	39.32±9.50	87.48±26.25	68.48±9.52	104.12±8.11	94.15±14.98	93.65±10.3
N-containing compounds (7)						
Trimethylamine	601.35±22.66	308.41±29.82	384.74±40.54	231.14±23.04	248.6±20.08	258.37±14.28
Dimethylamine	98.83±16.94	3.02±1.72	70.59±0.66		8.72±7.48	1.3±0.00
Acetamide	3.97±1.53	2.41±0.71	4.79±0.23	3.55±0.30	3.28±0.49	2.75±0.19
1-Methyl-1H-pyrrole	3.46±2.29	13.22±2.01	1.55±0.40	ND	ND	ND
2-Acetyl-1-pyrroline	7.45±2.92	ND	9.69±1.02	ND	6.19±0.23	8.22±0.53
2-Acetyl-pyridine	1.90±0.09	ND	ND	ND	ND	0.06±0.00
2-Acetylthiazole	5.08±1.54	4.04±1.70	5.65±2.15	5.70±0.44	6.99±3.46	5.08±1.54
subtotal	722.04±47.97	331.1±35.96	477.01±45	240.39±23.78	273.78±31.74	275.78±16.54
Ketones (17)						
1-Octen-3-one	ND	1.49±0.00	ND	ND	ND	0.15±0.05
2,3-Dihydro-3,5-dihydroxy-6-methyl-4H-pyran-4-one	2.94±0.83	3.80±1.63	5.40±0.08	3.88±0.68	4.80±2.03	2.76±0.94
Acetylbutyryl	ND	ND	ND	1.52±0.87	1.09±0.00	3.86±4.13
2-Pyrrolidinone	3.05±1.22	1.35±0.65	5.92±0.80	2.40±0.20	1.8±0.50	1.77±0.21
2-Butanone	12.46±1.19	1.60±0.00	ND	ND	ND	ND
2-Heptanone	6.52±0.93	19.51±2.35	6.66±0.70	6.87±0.30	7.25±2.22	7.98±1.92
2-Piperidinone	26.98±2.86	2.01±0.59	5.98±0.57	3.27±0.09	2.36±0.72	1.71±0.36
2-Nonanone	15.05±6.96	5.33±1.46	5.56±3.30	5.60±0.14	3.87±0.29	5.09±0.30
2-Tridecanone	0.57±0.02	ND	0.64±0.00	ND	0.58±0.00	ND
2-Undecanone	0.95±0.56	ND	1.84±0.28	1.46±0.53	0.80±0.34	2.09±0.00
2-Pentadecanone	0.62±0.20	0.58±0.03	0.74±0.00	0.58±0.07	1.01±0.00	0.75±0.26
2-Pentanon	ND	3.79±0.00	ND	ND	11.24±2.30	7.78±0.19
2-Octanone	6.15±0.98	4.82±2.88	5.21±0.73	3.06±0.20	3.20±0.62	3.11±0.15
3-Hexanone	ND	5.19±0.56	ND	ND	29.34±2.34	ND
6-Methyl-5-hepten-2-one	8.58±1.00	20.91±2.35	ND	12.07±0.50	7.17±0.65	19.83±0.99
Acetophenone	ND	ND	ND	9.33±1.09	ND	ND
Trans-geranylacetone	9.52±2.78	4.21±1.96	3.25±0.79	3.37±0.57	7.13±1.63	11.92±0.94
Subtotal	93.39±19.53	73.1±14.46	41.2±7.25	53.41±5.24	81.64±13.64	68.65±10.39
Alcohols (7)						
1-Tridecanol	2.71±1.46	2.12±0.19	1.48±0.00	1.41±0.00	1.68±0.00	2.71±1.46
1-Penten-3-ol	4.31±0.53	11.82±1.11	2.90±0.37	4.96±1.53	7.66±2.22	4.79±0.00
Benzyl alcohol	2.60±0.16	1.45±0.31	3.4±0.20.	6.27±0.40	1.81±0.07	1.62±0.12
1-Hexadecanol	1.31±0.00	ND	0.65±0.00	0.91±0.06	2.1±0.00	ND
1-Octanol	0.40±0.00	ND	ND	ND	0.33±0.00	ND
2-Ethylhexanol	ND	ND	ND	1.67±0.37	0.69±0.00	ND
1-Dodecanol	0.66±0.00	ND	ND	ND	1.78±0.00	2.32±0.00
Subtotal	11.99±2.15	15.39±1.61	8.43±0.37	15.22±2.36	16.05±2.29	11.44±1.58
Acids (11)						
2-Methylbutanoic acid	ND	ND	ND	ND	ND	19.39±3.98
2-Diethylacetic acid,	ND	3.29±2.41	4.70±0.93	10.76±1.47	5.09±1.42	7.75±1.31
2-Ethylhexanoic acid	0.79±0.34	ND	ND	0.46±0.00	ND	ND
3-Methybutanoic acidl	ND	ND	ND	ND	16.39±2.55	ND
3-Methylpentanoic acid	10.75±3.83	6.21±3.92	8.96±0.68	9.66±0.35	ND	ND
Benzoic acid	3.64±2.12	4.35±0.64	1.73±0.23	5.28±0.61	1.94±0.11	1.96±0.02
Decanoic acid	4.01±1.35	1.88±0.64	1.90±0.26	ND	2.78±0.00	2.14±0.00
Nonanoic acid	ND	ND	ND	ND	2.78±0.26	2.47±0.00
Hexadecanoic acid	6.83±2.21	5.31±2.46	2.77±0.69	1.29±0.20	ND	3.15±0.51
Octanoic acid	0.92±0.16	1.71±0.17	0.71±0.15	1.41±0.36	1.84±1.12	ND
Tetradecanoic acid	3.75±1.53	5.16±1.67	2.24±0.79	2.42±0.85	4.42±3.36	3.33±1.01
Subtotal	30.69±11.54	27.91±11.91	23.01±3.73	31.28±3.84	35.24±8.82	40.19±6.83
Hydrocarbons (11)						
2,4-Dimethyl-decane	ND	46.11±5.21	ND	ND	5.87±0.61	5.04±0.74
2,5,5-Trimethylheptane	ND	13.02±7.68	4.14±0.64	1.55±0.00	ND	5.78±0.54
2,6,10-Trimethyltridecane	6.03±2.19	0.83±0.10	3.37±0.29	3.61±0.17	1.6±0.00	ND
3,5,5-Trimethyl-2-hexene	2.65±0.00	1.80±0.00	ND	ND	3.04±0.13	3.02±0.20
3,7-Dimethyldecane	0.73±0.00	7.70±4.55	1.51±0.00	2.04±1.66	8.78±0.56	1.44±0.61

Decane	2.12±0.04	20.42±2.01	6.10±0.54	ND	14.64±0.95	7.01±3.27
Dodecane	2.61±1.45	14.01±8.04	4.98±0.28	8.62±9.66	8.12±0.99	6.33±3.64
Tridecane	1.82±1.18	8.37±1.30	4.52±0.00	7.96±1.99	1.26±1.05	9.66±1.24
Tetradecane	1.50±0.86	ND	0.32±0.00	1.00±0.00	1.24±0.40	0.93±0.00
Pentadecane	3.17±1.94	2.00±0.00	ND	0.96±0.00	0.59±0.00	0.71±0.06
Undecane	1.85±0.87	3.21±0.63	1.69±0.11	12.09±2.35	ND	1.87±0.25
Subtotal	22.48±8.53	117.47±29.52	26.63±1.86	37.83±15.83	45.14±4.69	41.79±10.55
Total	2014.22±156.69	1246.83±174.65	2041.52±145.09	1072.91±132.46	1135.29±126.38	1117.19±115.45

ND = not determined.
